# Human Sensorimotor Beta Event Characteristics and Aperiodic Signal Are Highly Heritable

**DOI:** 10.1523/JNEUROSCI.0265-23.2023

**Published:** 2024-01-31

**Authors:** K. Amande M. Pauls, Elina Salmela, Olesia Korsun, Jan Kujala, Riitta Salmelin, Hanna Renvall

**Affiliations:** ^1^ Department of Neurology, Helsinki University Hospital, and Department of Clinical Neurosciences, University of Helsinki, 00029 Helsinki, Finland; ^2^ BioMag Laboratory, HUS Medical Imaging Center, Helsinki University Hospital, 00290 Helsinki, Finland; ^3^ Organismal and Evolutionary Biology Research Programme, Faculty of Biological and Environmental Sciences, University of Helsinki, 00014 Helsinki, Finland; ^4^ Department of Biology, University of Turku, 20014 Turku, Finland; ^5^ Department of Neuroscience and Biomedical Engineering, School of Science, Aalto University, 02150 Espoo, Finland; ^6^ Department of Psychology, University of Jyväskylä, 40014 Jyväskylä, Finland

**Keywords:** 1/f (aperiodic) component, beta oscillation, heritability, magnetoencephalography, resting state, sensorimotor

## Abstract

Individuals’ phenotypes, including the brain's structure and function, are largely determined by genes and their interplay. The resting brain generates salient rhythmic patterns that can be characterized noninvasively using functional neuroimaging such as magnetoencephalography (MEG). One of these rhythms, the somatomotor (rolandic) beta rhythm, shows intermittent high amplitude “events” that predict behavior across tasks and species. Beta rhythm is altered in neurological disease. The aperiodic (1/f) signal present in electrophysiological recordings is also modulated by some neurological conditions and aging. Both sensorimotor beta and aperiodic signal could thus serve as biomarkers of sensorimotor function. Knowledge about the extent to which these brain functional measures are heritable could shed light on the mechanisms underlying their generation. We investigated the heritability and variability of human spontaneous sensorimotor beta rhythm events and aperiodic activity in 210 healthy male and female adult siblings’ spontaneous MEG activity. The most heritable trait was the aperiodic 1/f signal, with a heritability of 0.87 in the right hemisphere. Time-resolved beta event amplitude parameters were also highly heritable, whereas the heritabilities for overall beta power, peak frequency, and measures of event duration remained nonsignificant. Human sensorimotor neural activity can thus be dissected into different components with variable heritability. We postulate that these differences partially reflect different underlying signal-generating mechanisms. The 1/f signal and beta event amplitude measures may depend more on fixed, anatomical parameters, whereas beta event duration and its modulation reflect dynamic characteristics, guiding their use as potential disease biomarkers.

## Significance Statement

The resting brain shows a prominent, highly modulated beta-range rhythm closely linked to sensorimotor function in health and disease. We investigated the heritability of human spontaneous sensorimotor beta rhythm and its different components in a large cohort of 210 siblings’ magnetoencephalography (MEG) data. We find that, particularly, beta event amplitude and its variation, as well as aperiodic signal characteristics, are highly heritable. The study demonstrates that time-resolved electrophysiological measures of spontaneous human sensorimotor brain activity are determined to a significant degree by genes. We discuss the findings in the context of known and postulated structural underpinnings of MEG signal generation, to highlight their translational relevance. The findings have clinical implications, for example, when considering sensorimotor beta alterations as biomarkers of neurological disease.

## Introduction

Individuals’ phenotypes are largely determined by their genetic blueprint that regulates properties ranging from cell products (Barroso and McCarthy, 2019) to system-level brain macrostructure (Geschwind et al., 2002; Peper et al., 2007). Genetic influences also underlie functional brain measures which are constant within, but highly variable between individuals. Electroencephalography (EEG) and magnetoencephalography (MEG) have been successfully applied to quantify the heritability and identify genetic determinants of functional brain measures (Van Beijsterveldt et al., 1996; Smit et al., 2006; Koten et al., 2009; Renvall et al., 2012; van Pelt et al., 2012).

The brain generates “background” electrical activity with salient rhythmic, but also arrhythmic patterns during wakeful resting. One of the prominent spontaneous rhythms is the somatomotor (rolandic) beta rhythm (Hari and Salmelin, 1997) that is observed across several mammalian species (Haegens et al., 2011; Feingold et al., 2015; Sherman et al., 2016). It is modulated by perceptual and cognitive functions, including tactile processing (Pfurtscheller et al., 2001; Haegens et al., 2011), motor function (Salmelin and Hari, 1994; Feingold et al., 2015), action perception (Hari et al., 1998; Babiloni et al., 2002), and attention (Van Ede et al., 2011; Sacchet et al., 2015). Beta band activity is modulated over time, manifesting in intermittent high amplitude “events” (Feingold et al., 2015; Jones, 2016) relevant for behavior: In the sensorimotor cortex, beta event rate predicts behavior across tasks and species (Shin et al., 2017). Both beta power and beta events are altered in neurological conditions affecting motor function, such as genetically determined Unverricht–Lundborg disease (Silén et al., 2000), stroke (Laaksonen et al., 2012), and Parkinson's disease (Vinding et al., 2020; Pauls et al., 2022).

Besides rhythmic, or periodic, components, MEG power spectra also contain aperiodic (1/f) components (He, 2014). These two are important to disentangle as they are probably generated by different neural mechanisms. Aperiodic signal is believed to represent excitation–inhibition balance (Gao et al., 2017), and it is modulated, for example, by brain maturation (McSweeney et al., 2021; Hill et al., 2022), aging (Voytek et al., 2015; Wilson et al., 2022), and several neurological and psychiatric conditions (Molina et al., 2020; Ostlund et al., 2021; Semenova et al., 2021). Cortical beta rhythm (Laaksonen et al., 2012; Pauls et al., 2022) and aperiodic activity (Helson et al., 2023) both relate to clinical symptoms, show good or excellent test–retest reliability (Pauls et al., 2023), and thus have potential as diagnostic or prognostic biomarkers.

Interpretability of rhythmic and aperiodic neural signals is important for both research and clinical diagnostic applications. MEG signal arises from spatial and temporal summation of underlying neuronal activity (Buzsáki et al., 2012). Structure and function are closely related: for example, peak oscillation frequency decreases with increasing cortical thickness and processing hierarchy (Mahjoory et al., 2020). Decoding the structure–function–genetics relationship of M/EEG signal generation could help understand signals’ individuality and their degradation in neurological diseases, raising their value as diagnostic tools: M/EEG may detect pathology before observable structural changes in neurological disorders (Terry et al., 1991). Heritability reflects the contribution of genetic versus environmental factors to the differences observed between individuals, and the quantification of the heritability of neural signals can thus lead to insights of the biology behind the measurable phenotypes (Visscher et al., 2008). Beta and other frequency bands’ global spectral power is heritable (Van Baal et al., 1996; Van Beijsterveldt et al., 1996; Smit et al., 2005; Salmela et al., 2016); the beta power variability has been linked to a GABA_A_ receptor locus (Porjesz et al., 2002). Heritability of time-resolved beta events, however, has not been investigated.

We investigated the heritability and variability of time-resolved human cortical sensorimotor beta rhythm and aperiodic activity using healthy adult siblings’ spontaneous MEG data. We propose that knowledge about the relative heritability of different neural components of sensorimotor activity can shed light on the underlying generating mechanisms and help interpret changes observed in, for example, patient populations with sensorimotor dysfunction.

## Materials and Methods

### Subjects

Two hundred ten Finnish-speaking siblings from 100 families participated in the study (8 families with three siblings, 1 family with four; 148 females [mean ± SD age 29 ± 10 years, range 18–60 years], 62 males [30 ± 9 years, range 19–52 years]; 206 right-handed, three ambidextrous, one left-handed). None of the participants had a history of neurological or psychiatric disorders. The study was approved by the Hospital District of Helsinki and Uusimaa ethics committee, and all participants gave their written informed consent to participate.

### MEG recordings

Spontaneous cortical activity was recorded in a magnetically shielded room with a 306-channel Vectorview neuromagnetometer (Elekta Oy) that contains 204 planar gradiometers and 102 magnetometers. Head positioning was measured at the beginning of the measurement. Three minutes of data were collected while participants were resting with their eyes open (REST), as well as while they clenched both hands alternatingly about once per second, self-paced, keeping the eyes open (MOT). The MEG signals were bandpass filtered at 0.03–200 Hz and sampled at 600 Hz.

### MEG signal processing and beta event extraction

For suppressing external artifacts, MEG data were preprocessed using the signal space separation method (SSS; Taulu and Simola, 2006) implemented in MaxFilter software (MEGIN Oy). Individual MEG recordings were transferred to one subject's head space using a signal space separation-based head transformation algorithm (Taulu et al., 2004), implemented in MaxFilter. Further signal processing was done using MNE-Python version 0.22 (Gramfort et al., 2013). After bandpass filtering the data to 2–48 Hz with a one-pass, zero-phase, noncausal FIR filter (MNE firwin filter design using a Hamming window), power spectral density (PSD) was calculated using Welch's method (MNE's psd_welch function) with a nonoverlapping Hamming window and 1024-point fast Fourier transformation (FFT).

**Figure 1.**
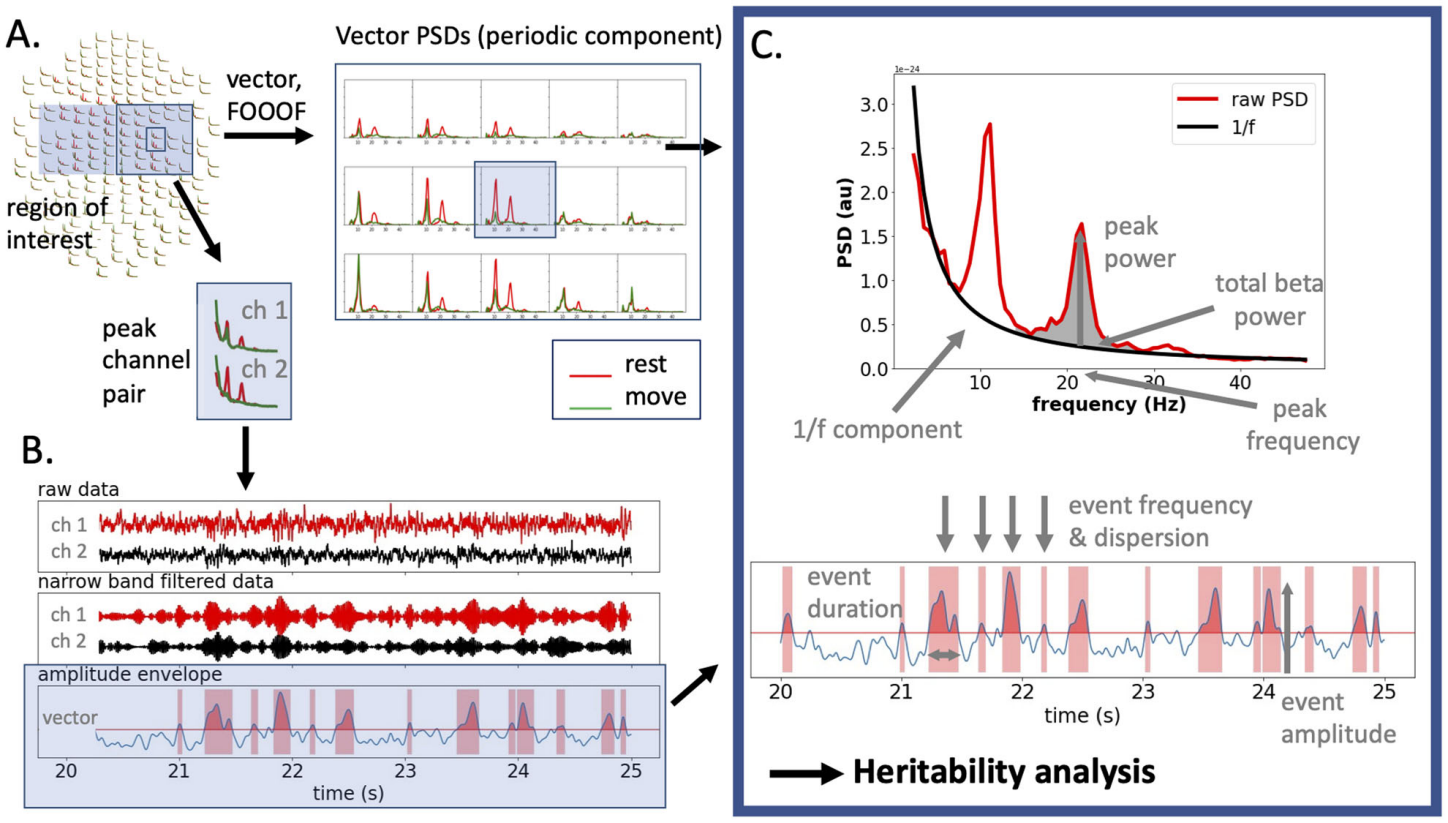
Extraction of sensorimotor beta phenotype characteristics. ***A***, Channel selection. A ROI was defined for both hemispheres. The 15 selected gradiometer-channel pairs were combined into 15 vector-sum PSDs (one per channel pair). The periodic spectral component of the vector-sum PSD was obtained using FOOOF. From these, a peak beta frequency and peak channel pair were selected. ***B***, Beta event extraction. The peak channel pair and peak frequency selected in ***A*** were used to calculate the channel pair's amplitude envelope. From the raw data, narrow-band-filtered data were obtained using wavelet decomposition, and the individual channels’ band-filtered signals were combined to one amplitude envelope using vector sum calculation. ***C***, Parameters for heritability analysis. Both PSD characteristics [beta peak power and frequency, total beta power at 14–30 Hz (periodic part), 1/f exponent; top panel] and time-resolved beta oscillatory characteristics (beta events; bottom panel) were used in the heritability analysis.

The subsequent analysis steps are illustrated in Figure 1. The data analysis was performed on the 204 gradiometer signals. First, a channel pair with the highest spectral peak in the beta range (the peak channel pair) was selected from the region of interest (ROI) of 15 gradiometer-channel pairs per hemisphere centered over the sensorimotor cortices, and the frequency at the power peak noted (peak beta frequency) (Fig. 1*A*). In order to quantify PSD at each recording site, we computed the vector sum of the two orthogonally oriented planar gradiometers at each sensor location (vector PSD):

$${\rm PSD_{vector}} \equals \surd \lpar {{\rm PS}{\rm D}_{{\rm ch}1}^2 \plus {\rm PS}{\rm D}_{{\rm ch}2}^2 } \rpar.$$

The resulting 15 vector-sum PSDs per hemisphere were then decomposed into a periodic and aperiodic component using FOOOF (Donoghue et al., 2020). FOOOF models the power spectrum as a combination of two distinct functional processes: an aperiodic component, reflecting 1/f-like characteristics (exponential decay with an offset and an exponent), and a variable number of periodic components (putative oscillations), as peaks rising above the aperiodic component. After subtraction of the aperiodic component, the remaining periodic component was plotted for all 15 vector-sum PSDs for both REST and MOT conditions in the frequency range of 14–30 Hz. The resulting plots were visually inspected by two observers (K.A.M.P. and O.K.) to manually select the beta signal frequency modulated most by MOT compared to REST.

As the manual channel selection may be prone to human observer bias, we compared the inter-rater agreement between two slightly different approaches, conducted independently years apart on the same data. The peak beta band frequencies had previously been extracted by one of the authors (H.R.) without separating the 1/f aperiodic signal part and by using Welch's method with 4096-point FFT, eight data segments overlapping by 50% and Hamming windowing. When allowing deviation of ±3 Hz in the extracted peaks (taken the different FFT sizes and different handling of the aperiodic 1/f component), the two approaches resulted in 85% agreement, which is considered good.

Using the manually selected peak frequencies, the periodic components of the 15 vector-sum PSDs were searched automatically to determine the recording channel with the highest peak and its frequency (±1 Hz) for both hemispheres’ ROIs, and visually inspected again by K.A.M.P.

The peak beta frequency and corresponding peak power of the chosen vector-sum PSD, the total beta band power (periodic part of PSD area under curve (AUC) from 14 to 30 Hz, 1/f component subtracted), as well as the aperiodic component information obtained via FOOOF (offset and exponent chi), were further used in the heritability analysis. All electrophysiological parameters included in the heritability analysis are illustrated in Figure 1*C*.

The channel pair and peak beta frequency corresponding to the chosen vector-sum PSD were used for beta burst analysis (Fig. 1*B*). Beta event extraction was carried out similarly to the method described in Pauls et al. (2022): the channel pair's raw unfiltered time series data were downsampled to 200 Hz, high-pass filtered at 2 Hz and decomposed by convolving the signal with a set of complex Morlet wavelets over the frequency range of 7–47 Hz with 1 Hz resolution and n_cycles = frequency/2. The signal was then averaged within the individual narrow-band beta frequency range, that is, ±1.5 Hz around the individual peak beta frequency, discarding the other frequencies. The vector sum over the two channels’ beta band time series was calculated as described above, and the resulting signal was rectified to obtain one beta band amplitude envelope for the channel pair. The envelope was smoothed with a 100-ms FWHM kernel and thresholded at the 75th percentile value. Periods exceeding this threshold for 50 ms or longer were defined as beta events. Beta event parameters are illustrated in Figure 1*C*. For event amplitude and event duration, the mean, median, robust maximum (defined as mean of the top 5% values), and standard deviation values were calculated. Furthermore, events per second (event rate) and event dispersion were calculated similarly to Pauls et al. (2022). Times between beta events were defined as waiting times. To estimate the variation of waiting times (event dispersion), we calculated the coefficient *C_V_* proposed by Shinomoto et al. (2005), defined as the waiting times’ standard deviation σ divided by their mean μ:

$$C_V \equals \displaystyle{\sigma \over \mu }.$$

All values were calculated for both hemispheres in all subjects (Fig. 1*C*).

Effect sizes for MEG features were based on Cohen's *d* values for single-group designs:

$$D \equals M{\rm \sol }S,$$

where *M* and *S* are the mean and the standard deviation of the feature values across subjects (Goulet-Pelletier and Cousineau, 2018).

### Heritability analysis

Heritability is defined as the proportion of (additive) genetic variance of the total phenotypic variance of a population.

$$h^2 \equals V_{{\rm genetic}}{\rm \sol }V_{{\rm phenotypic}}.$$

Phenotype heritabilities were calculated using the software program Merlin version 1.1.2 (Abecasis et al., 2002), which employs a variance component approach as detailed by Amos (1994). Heritability estimates are calculated based on variance components. The coefficient estimating genetic variance is adjusted by the degree of relationship, which is 0.5 (50% shared genes) in full siblings. The full sibling status of our study individuals has been confirmed by [an earlier] DNA analysis (Renvall et al., 2012).

Merlin requires nonnegative values for correct interpretation, so phenotypes with negative values were multiplied by −1. Such a transformation is standard for the Merlin analysis tool. Correctness of the input data format was checked by the Pedstats program (Wigginton and Abecasis, 2005). As the analysis assumes the studied phenotypes to be normally distributed while many of them were not, we also reran the analyses after first correcting the phenotype values’ distributions with the inverse normal correction internal to Merlin. As both analyses produced highly concordant results, we report here the results based on the noncorrected values.

The probability of the observed heritability values being different from zero was assessed by permuting the family labels of the study subjects 6,000 times and calculating the heritability for each of the permuted datasets. For each phenotype, the number of permutations *k* where the permuted heritability was higher than the heritability observed in the real data was recorded and used to calculate the one-tailed probability of the observed heritability exceeding zero as *k*/6,000. This permutation scheme may slightly inflate the permuted heritabilities, as it does not explicitly ensure that the permutation does not reproduce any of the original sibships. This may lead to conservative significance estimates. Likewise, to correct for the multiple tests performed (*n* = 30), we performed a Bonferroni correction, which may be overly conservative considering that some of the phenotypes were correlated.

### Code and data accessibility

These data cannot be made publicly available due to Finnish data protection law. Data can, however, be shared for research collaboration with an amendment to the research ethics permit and a related data transfer agreement. All analysis code is available on GitHub (https://github.com/BioMag/Beta-sibling-study).

## Results

**Figure 2.**
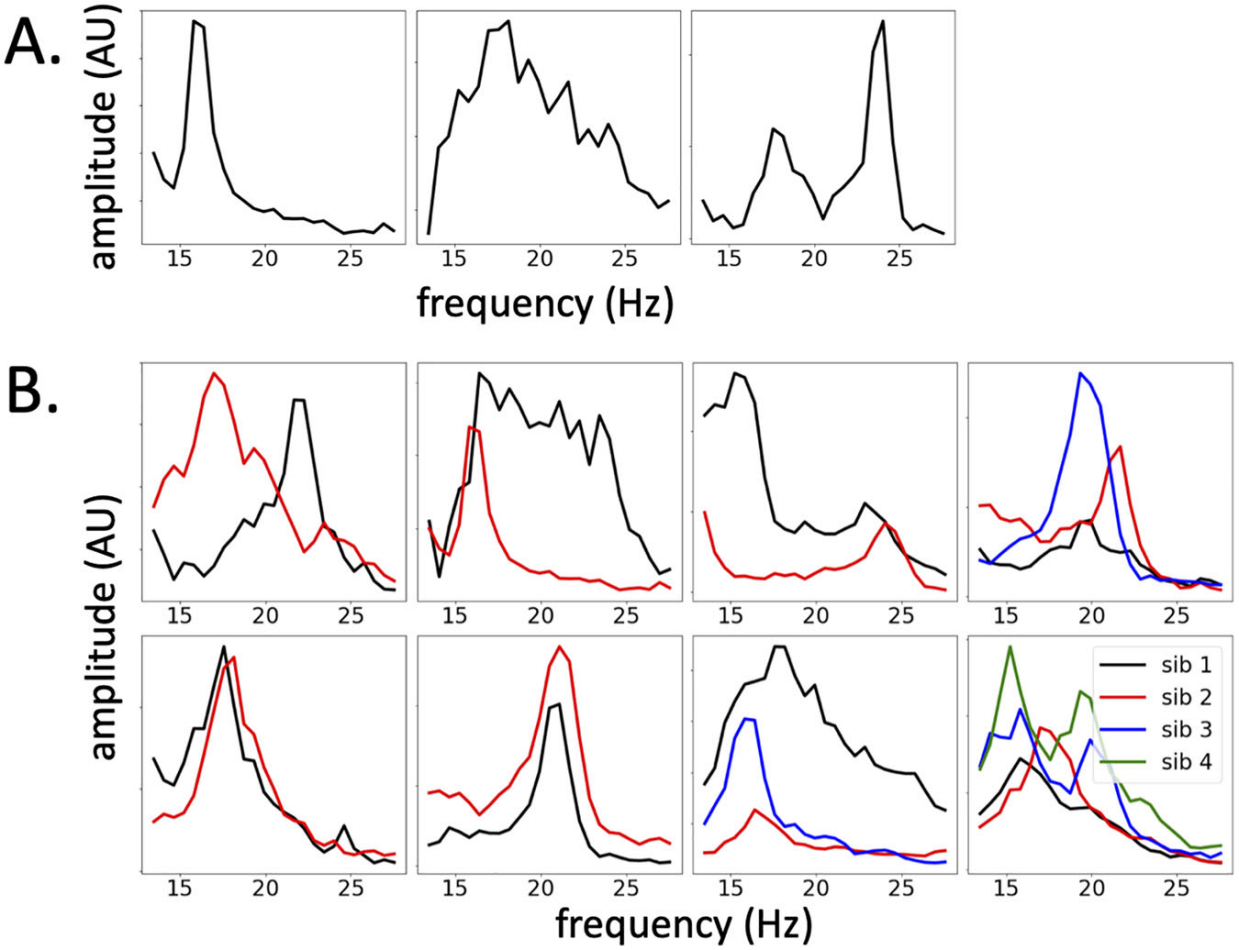
Beta phenotypes. ***A***, Phenotypic spectrum of beta activity. Examples of typical beta range PSD patterns: left - narrow beta peak, middle - broad range, “beta brush” like activity, right - double peaks of comparable strength, one in the lower, one in the higher beta range. ***B***, Beta PSD patterns in siblings. Examples of siblings’ beta PSD patterns (two families with two siblings, one family with three siblings, one family with four siblings).

A summary of the beta band phenotypic features (both beta PSD features as well as beta band burst characteristics) is given in Table 1. Figure 2*A* shows examples of different beta power spectral phenotypes observed, and Figure 2*B* depicts beta band phenotypes for pairs of siblings. Typical PSD phenotypes were (1) ones with a narrow peak on either side of 20 Hz, (2) a broad band activity typically spanning 15–25 Hz, and (3) two distinctive peaks, one typically in the lower beta range (14–20 Hz) and the other in the high beta range (20–30 Hz).

**Table 1.** PSD (beta and 1/f) and beta event descriptives

		Left hemisphere				Right hemisphere			
		Mean	Median	SD	Range	Mean	Median	SD	Range
*PSD characteristics*									
Peak beta frequency (Hz)		19.7	19.3	3.0	14.1–25.8	19.8	19.3	3.1	14.1–29.3
Peak beta power (fT/cm)^2^		276	144	374	15–3,231	133	70	173	5–1,251
Total beta band power (periodic)		2,979	1,686	3,318	164–16,284	1,093	619	1,322	26–9,652
1/f component exponent		1.03	1.01	0.19	0.34–1.76	1.07	1.05	0.18	0.67–1.66
1/f component offset		−22.95	−22.99	0.37	−23.75 to −21.75	−23.28	−23.32	0.34	−24.19 to −22.25
*Beta event characteristics*									
Duration (ms)	Mean	256.9	248.0	49.4	181.7–498.2	265.2	253.7	50.0	182.0–454.0
	Median	199.0	195.0	32.3	152.5–420.0	201.4	195.0	34.9	150.0–355.0
	Standard deviation	198.4	182.4	63.2	99.8–487.5	213.0	198.3	67.4	67.3–531.7
	Robust maximum	858.0	788.6	257.4	439.5–2,145.0	912.1	852.2	263.9	382.7–2,135.0
Amplitude (fT/cm)	Mean	325	285	165	104–994	221	188	113	73–654
	Median	301	260	155	93–984	203	173	105	70–618
	Standard deviation	86	71	48	21–264	62	52	34	14–202
	Robust maximum	564	486	286	174–1,467	390	335	196	114–1,128
Event rate (1/s)		1.00	1.00	0.16	0.50–1.36	0.97	0.98	0.16	0.55–1.37
Dispersion		1.14	1.05	0.44	0.65–5.59	1.16	1.06	0.44	0.41–5.58

Parameters used in the heritability analysis. Peak frequency—frequency between 14 and 30 Hz most modulated by hand movement; peak power—PSD amplitude at peak frequency; total beta band power (periodic)—total AUC from 14 to 30 Hz of the periodic part of the signal (1/f signal component subtracted); 1/f component chi—exponential decay coefficient and offset describing 1/f (aperiodic) signal component. Beta event characteristics: robust maximum—mean of top 5% values; burst rate—number of bursts/recording time; dispersion—SD(inter-burst intervals)/mean(inter-burst intervals).

Heritability results are shown in Table 2. Overall, the right-hemispheric parameters were more heritable than the left-hemispheric ones. The right hemisphere's 1/f aperiodic exponent and offset were significantly heritable (exponent *h*^2 ^= 0.87, offset *h*^2 ^= 0.69). Measures of beta burst amplitudes were also significantly heritable (range of significant heritability values *h*^2^ of 0.28–0.81). Notably, of the beta burst amplitude measures, the measures reflecting the dynamic range (beta event amplitude maximum and its standard deviation) were most highly heritable. Apart from the peak beta power with moderate effect size in both hemispheres (Cohen's *d* = 0.74–0.77), all effect sizes were either large (Cohen's *d *> 0.80) or very large (Cohen's *d* > 1.2).

**Table 2.** Heritability *h*^2^ of the oscillatory phenotypes calculated by Merlin

		Left hemisphere			Right hemisphere		
		*h* ^2^	*p*	*n* sig. (/6,000)	*h* ^2^	*p*	*n* sig. (/6,000)
*PSD characteristics*							
Peak beta frequency		0.45	0.0047	28	0.41	0.0103	62
Peak beta power		0.28	0.0648	389	0.58	0.0072	43
Total beta band power (periodic)		0.49	0.0068	41	0.44	0.0157	94
1/f component exponent*		0.47	0.0035	21	**0.87**	0.0000*	**0**
1/f component offset*		0.35	0.0258	155	**0.69**	0.0000*	**0**
*Beta event characteristics*							
Duration	Mean	0.45	0.1350	810	0.36	0.0222	133
	Median	0.28	0.1338	803	0.40	0.0172	103
	Standard deviation	0.49	0.2495	1,497	0.32	0.0412	247
	Robust maximum	0.47	0.2383	1,430	0.33	0.0372	223
Amplitude	Mean*	0.35	0.0060	36	**0.75**	0.0002*	**1**
	Median*	0.45	0.0110	66	**0.72**	0.0002*	**1**
	Standard deviation*	**0.28**	0.0005*	**3**	**0.81**	0.0000*	**0**
	Robust maximum*	**0.49**	0.0007*	**4**	**0.79**	0.0000*	**0**
Event rate		0.47	0.0543	326	0.38	0.0137	82
Dispersion		0.35	0.2850	1,710	0.00	1.0000	6,000

The nominal probability that the heritability differs from zero is calculated from an empirical distribution based on 6,000 permutations of the sibship statuses/family IDs of the subjects. The variables and values that are significant after a Bonferroni correction for multiple testing are given in bold. * indicates variables and values with significant heritability findings.

## Discussion

To our knowledge, this is the first study investigating the heritability of spontaneous time-resolved sensorimotor beta event dynamics and aperiodic neural activity. Time-resolved beta event amplitude parameters were highly heritable, whereas the heritabilities for peak frequency and measures of event duration were not significantly different from zero. Interestingly, the most heritable trait was the aperiodic 1/f exponent, with a heritability of 0.87 in the right hemisphere. Overall, the right-hemispheric phenotypic traits were more heritable than the left-hemispheric ones.

### Heritability of MEG/EEG traits including beta oscillatory activity

Heritability of electrophysiological traits has been little investigated to date. In twin studies, EEG alpha, beta, theta, and delta range peak frequencies (Van Beijsterveldt et al., 1996), occipital alpha power and peak frequency at rest (Smit et al., 2006), as well as MEG visual task-related gamma peak frequency (van Pelt et al., 2012) have been found to be highly heritable. We have previously demonstrated that auditory evoked fields’ amplitude (Renvall et al., 2012), as well as occipital resting-state alpha oscillatory activity (Salmela et al., 2016), are heritable in siblings and that MEG power spectral features at rest allow identification of sibling relationship (Leppäaho et al., 2019). These MEG traits were associated with certain genetic loci/genomic regions (Renvall et al., 2012; Salmela et al., 2016; Leppäaho et al., 2019) but it is likely that most functional brain traits are controlled polygenetically. Furthermore, functional connectivity in theta, alpha and beta bands as measured with MEG appears progressively more similar as the strength of the genetic relationship increases (Colclough et al., 2017).

### MEG signal generative mechanisms and possible relation to heritability

**Figure 3.**
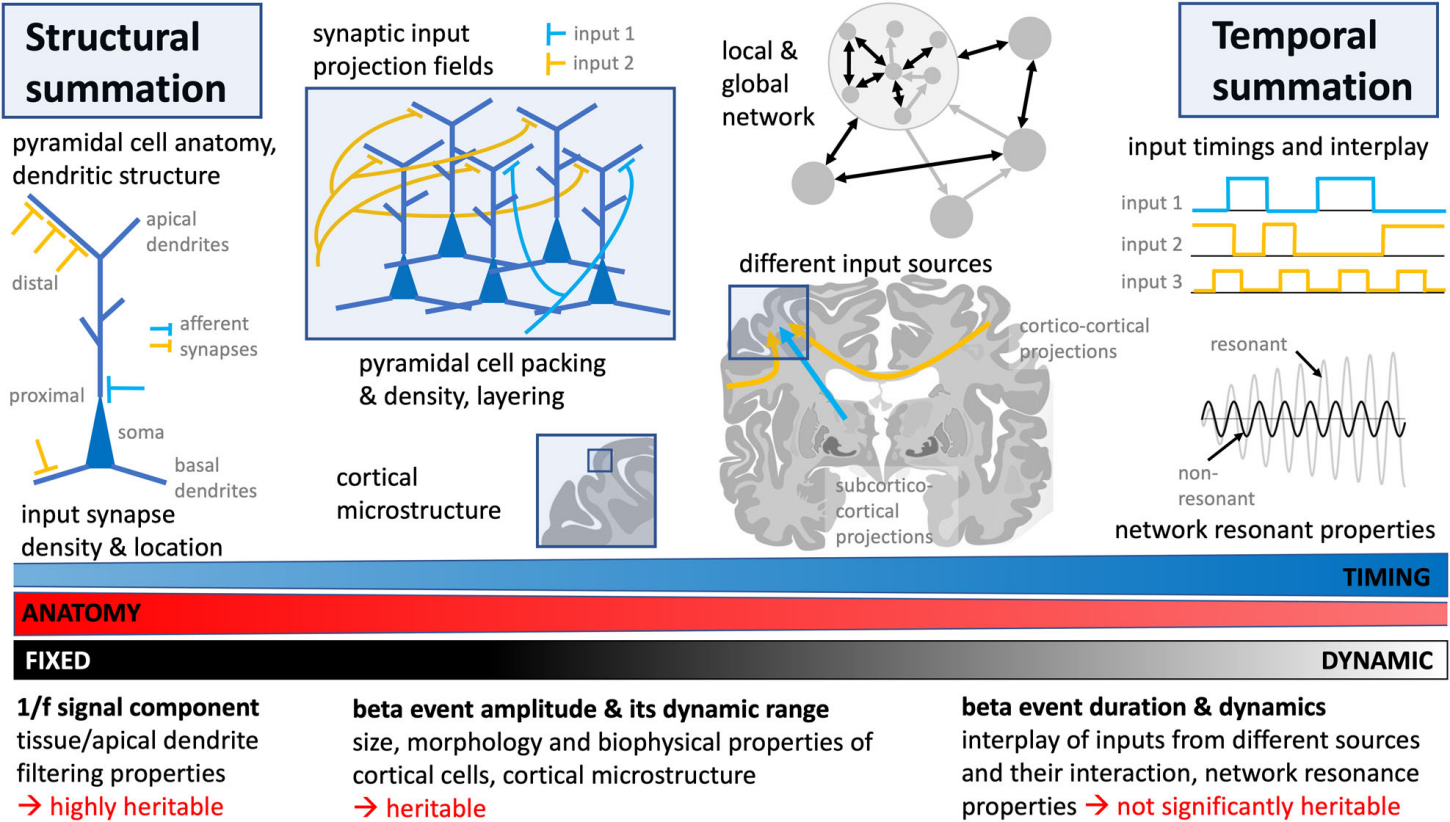
Sources of MEG signals and their putative relationship to the MEG parameters examined in the present study. The schematic figure's top panel summarizes the main anatomical and morphological factors, as well as factors determining timing of events, that contribute to the generation of MEG signals. The bottom part indicates the putative relationship of those factors to the MEG parameters examined here. We postulate that the 1/f signal and beta event amplitude parameters are more heavily dependent on fixed, anatomical parameters, whereas beta event duration and its modulation are more dynamic characteristics, yet keeping in mind that timing is very much constrained by network anatomy. Brain slice modified from: https://commons.wikimedia.org/wiki/File:Human_basal_ganglia_nuclei_as_shown_in_two_coronal_slices_and_with_reference_to_an_illustration_in_the_sagital_plane.svg.

MEG measures magnetic fields arising from the *temporal* and *spatial summation* of electric currents occurring in the underlying brain tissue (Buzsáki et al., 2012). The measured raw signal time series can be summarized in different ways, for example, as PSD. Reduction in global beta power can result from various changes in the neuronal signaling, such as smaller amplitude beta oscillation events, or fewer or shorter beta oscillation events without simultaneous changes in amplitude. Thus, decomposing beta power into components gives additional information about the underlying neural processing. We postulate that these MEG dynamical measures reflect different aspects of MEG signal generation. The top panel of Figure 3 schematically summarizes factors that contribute to the generation of MEG signals, and the bottom panel indicates how those factors may relate to the functional parameters addressed in this study.

### What underlies the heritability of beta event amplitude?

We postulate that the MEG beta event amplitude reflects relatively fixed anatomical factors summarized in Figure 3 (top panel, left). Pyramidal cells are neocortex’ most abundant cell type. Synaptic currents and their state-dependent modulation are the main determinants of intra- and extracellular field strength, and their *spatial summation* is governed by pyramidal cell morphology, cortical microstructure, and layering, as well as synaptic input density (Buzsáki et al., 2012). Beta event amplitudes are probably crucially dependent on these microstructural properties: While both temporal and spatial superposition determine event amplitude, especially the amplitude's dynamic range is limited by local cortical microstructure. Interestingly, in the current study, event amplitudes’ dynamic range measures (standard deviation, maximum) were most strongly heritable.

Brain anatomical traits such as cortical thickness (Geschwind et al., 2002; Schmitt et al., 2014) and cortical myelination (Schmitt et al., 2020) have previously been shown to be heritable. By late adolescence, differences in cortical thickness in the sensorimotor regions are largely due to heritable factors, whereas environmental factors play only a weak role (Schmitt et al., 2014). Thus, throughout development, sensorimotor cortical structure appears increasingly governed by the underlying genetics.

Both beta peak amplitude and the power at the beta band (which is determined by the amplitude, number, and duration of individual beta events) appeared more heritable in the right than the left hemisphere. Our result is in agreement with earlier studies that have found cortical morphology/volume to be more genetically controlled in the right than left hemisphere in right-handed individuals (Geschwind et al., 2002); functional studies point in the same direction (Smit et al., 2006).

### Why are event duration parameters not similarly heritable?

In the current cohort, measures of beta event duration were not significantly heritable. *Temporal summation* of neural events, which determines the timing and duration of beta events, arises from the interplay between several brain areas, their connections and relative input timings and strength (Fig. 3, top panel, right). Important cortical pyramidal cell afferent inputs originate from other adjacent pyramidal cells (intrinsic input) (Lorente de No, 1949), corticocortical connections (Kandel et al., 2000), and thalamic connections, including connections from sensory organs, and from other cortical areas (“higher-order” thalamic input) (Sherman et al., 2016; Mo and Sherman, 2019). Computational models suggest that sensory-induced beta events are generated by synchronous bursts of excitatory synaptic drive to superficial and deep cortical layers, with asymmetry in the respective input strengths (Jones et al., 2009; Sherman et al., 2016; Neymotin et al., 2020): The stronger the superficial input, the more prominent is the beta activity (Sherman et al., 2016). Experimental data are compatible with this model (Sherman et al., 2016; Bonaiuto et al., 2021; Law et al., 2022). Thus, beta event timing and duration appear to depend on the timing and strength of inputs from several different cortical and subcortical input sources.

Network resonance could also play a role in beta event generation: In a dopamine-depleted state, cortical beta events are associated with increased synchrony between EEG/ECoG cortical activity and basal ganglia spiking activity (Cagnan et al., 2019). In animal models of parkinsonism, high cortical beta synchrony can be generated by changing the relative timings between thalamic and corticocortical inputs (Reis et al., 2019). Hence, network resonant properties could contribute to temporal summation at least in some disease states, but possibly in a dopamine-dependent fashion also in healthy brains.

Thus, compared to spatial summation, temporal summation relies on more individual factors and their interplay (e.g., network structural and functional properties), making heritability more multifactorial and thus less likely to show heritability in the present analysis. Methodological factors could also contribute to the lack of heritability: signal-to-noise ratio of the recordings affects event duration more than event amplitude measures. Finally, the resting-state beta event duration could be a randomly fluctuating parameter, governed by stochastic events and their timing. These explanations, however, seem less likely given the outlined experimental evidence, as well as our test–retest reliability results (Pauls et al., 2023).

### Why is the aperiodic signal component heritable?

Aperiodic signal components were the most heritable of the investigated parameters in the present study. The aperiodic signal is closely related to anatomical microstructure: Cortical pyramidal cells and their dendritic morphology and density are believed to be the most important determinants of the mammalian cortical 1/f signal observed with MEG (Lindén et al., 2010; Buzsáki et al., 2012). The 1/f signal is thought to stem from passive dendrite filtering properties (Halnes et al., 2016) but it is also modulated in an activity-dependent way (Pettersen et al., 2014). It has been shown to be affected by brain maturation (McSweeney et al., 2021; Hill et al., 2022) and aging (Voytek et al., 2015; Wilson et al., 2022) as well as neurological (Semenova et al., 2021) and psychiatric diseases (Ostlund et al., 2021). Furthermore, 1/f reflects the attentional state (Waschke et al., 2021) and may contribute to the integration of signals over longer periods of time (Maniscalco et al., 2018). Thus, the signal's relative stability over extended periods of time, and its close relationship to cortical microstructure may explain the high heritability.

### Stability of beta events and aperiodic activity—a prerequisite for clinical use

Movement-related beta suppression and rebound at the sensorimotor cortices show excellent test–retest stability over weeks in EEG recordings (Espenhahn et al., 2017). Similarly, beta rhythm modulation after tactile and proprioceptive stimulation was recently demonstrated to be highly reproducible in healthy subjects within a year (Illman et al., 2022). In an independent cohort of 50 healthy subjects measured twice during wakeful resting, both the aperiodic power spectral features as well as several beta event characteristics showed good to excellent test–retest stability (Pauls et al., 2023). Recordings of 2–3 min of resting state data were sufficient to obtain stable results for most parameters, speaking for their feasibility in clinical settings. In the future, the heritability of dynamic oscillatory activity also outside the somatosensory cortices could be addressed. This would, however, likely require automated approaches which, in turn, might be more prone to signal-to-noise variations than the partly manual phenotyping applied here.

### Limitations

As the analysis assumes normal distribution of the phenotypes, the fact that many of the phenotypes were nonnormally distributed may have decreased the statistical power of the study. The permutation procedure adopted for testing the significance of the heritability values should, however, correct for any inflation of the heritabilities caused by the nonnormality. The analyses were additionally conducted with the internal normality correction functionality of Merlin, resulting in values qualitatively similar to (although slightly more significant than) those based on the noncorrected data presented here.

Any measurement noise contributes to the phenotypic variability, thus reducing estimated heritability. The effect sizes calculated here did not suggest a systematic effect of signal-to-noise ratio on the observed heritabilities: for example, the effect size for event duration was higher than the effect size for event amplitude. Furthermore, in our recent study (Pauls et al., 2023), the test–retest reliability of somatomotor beta activity was not directly related to relative heritabilities observed in the current study. Thus, the observed heritability differences do not solely reflect differences in the signal reliability or the signal-to-noise ratio.

## Conclusion

We here show that the human sensorimotor beta and aperiodic cortical activity can be dissected into highly heritable and nonheritable components. We postulate that the different heritabilities reflect, in part, different underlying signal-generating mechanisms and their weighting in the generation of different signal characteristics. In combination with increased information resulting from the time-resolved beta signal decomposition, the results generate an interesting framework to interrogate and interpret M/EEG data both in healthy subjects as well as patient populations. This framework also increases the potential of whole-brain electrophysiology measures, such as beta band activity, as disease biomarkers.
